# Canadine from *Corydalis turtschaninovii* Stimulates Myoblast Differentiation and Protects against Myotube Atrophy

**DOI:** 10.3390/ijms18122748

**Published:** 2017-12-18

**Authors:** Hyejin Lee, Sang-Jin Lee, Gyu-Un Bae, Nam-In Baek, Jae-Ha Ryu

**Affiliations:** 1 Research Center for Cell Fate Control and College of Pharmacy, Sookmyung Women’s University, 100 Chungparo 47-Gil, Yongsan-Gu, Seoul 04310, Korea; u9698115@naver.com (H.L.); lee.sangjin74@gmail.com (S.-J.L.); gbae@sookmyung.ac.kr (G.-U.B.); 2 The Graduate School of Biotechnology, Kyung Hee University, Yongin, Gyeonggi 17104, Korea; nibaek@khu.ac.kr

**Keywords:** *Corydalis turtschaninovii*, isoquinoline alkaloid, canadine, myoblast differentiation, muscle atrophy

## Abstract

Cachexia and sarcopenia are the main causes of muscle atrophy. These result in a reduction in the muscle fiber area, myo-protein content, and muscle strength, with various molecular modulators being involved. Although several reports have proposed potential therapeutic agents, no effective treatments have been found for muscle atrophy. We searched for myogenic modulators from medicinal plants to treat muscle diseases. We isolated six alkaloids from *Corydalis turtschaninovii* and evaluated their myogenic potential by using the MyoD reporter gene assay in C2C12 cells. Among the tested compounds, canadine showed the strongest transactivation of MyoD and increased MHC expression during myogenesis. The activation of p38 MAP kinase and Akt are major mechanisms that contribute to the myogenesis by canadine. Canadine increased the number of multinucleated and cylinder-shaped myotubes during myogenesis of C2C12 myoblasts. To determine the preventive effect of canadine in cancer-induced muscle wasting, differentiated C2C12 myotubes were treated with conditioned media from CT26 colon carcinoma culture (CT26 CM) in the presence of canadine. Canadine ameliorated the muscle protein degradation caused by CT26-CM by down-regulating the muscle specific-E3 ligases, MAFbx/atrogin-1 and MuRF1. In this study, we found that canadine from *C. turtschaninovii* stimulates myogenesis and also inhibits muscle protein degradation. Therefore, we suggest canadine as a protective agent against muscle atrophy.

## 1. Introduction

The loss of skeletal muscle mass is a common phenotype of cachexia and sarcopenia. This loss of muscle mass has a negative effect on the quality of life and survival rate of cancer patients. More than 30% of cancer patient mortality is a result of weight loss due to muscle impairment. This muscle atrophy is accompanied by a progressive decrease in muscle fiber cross-sectional area, muscle strength, nuclear number of myofibers and insulin responsiveness, together with the degradation of myo-proteins [1]. To overcome muscle wasting, we need to stimulate the myogenesis pathway or inhibit the muscle wasting process. The strategies to treat muscle wasting can be classified by action mechanism of drugs. We can regulate muscle wasting by down-regulating the inflammatory molecules and myostatin [2], or up-regulating cyclic AMP, proliferator-activated receptor gamma coactivator (PGC)-1α and the insulin signaling pathway [3]. These mechanisms contribute to an inhibition of the catabolism or activation of the anabolism of muscle proteins [4].

Five proteolytic systems are implicated in muscle atrophy, including the ubiquitin-proteasome system, caspase system, Ca^2+^-dependent calpain, Ca^2+^-independent cathepsin L, and autophagy. Recent reports demonstrated that the majority of potential drugs inhibit two or more proteolytic systems [5]. In addition, these drugs can overcome the muscle satellite dysfunction associated with muscle atrophy [6]. Therefore, we need to enhance the proliferation and differentiation of satellite cell-derived myoblasts to treat muscle atrophy.

Several natural products including citrus peel extract [7], salidroside [8], baicalin [9], ursolic acid [10], and tomatidine [11] were reported to ameliorate the muscle atrophy. We previously reported on several compounds as myogenic activators [12,13,14]. Despite long attempts to discover potential therapeutics to treat muscle atrophy, megestrol acetate (MA) is the only one approved by the US FDA to treat cancer and AIDS-induced cachexia.

In Oriental medicine, *Corydalis turtschaninovii* (CT) tubers have been used to treat inflammation, dysmenorrhea and allergic disease [15]. In Korea, the CT extract has been developed into a prokinetic drug (Motilitone^®^) by Dong-A Pharmaceutical Company. Recently, we reported the myogenic potential of dehydroxycorydaline and tetrahydropalmatine from CT [12,14]. Since CT contains isoquinoline alkaloids as main components, we tried to identify the most effective myogenic alkaloid, and to disclose its underlying mechanisms. In this study, we found that canadine was the most effective myogenic component of CT, and it prevented cancer cell-conditioned media-induced muscle wasting.

## 2. Results

### 2.1. Purification of Compounds from Corydalis turtschaninovii

**Figure 1:**
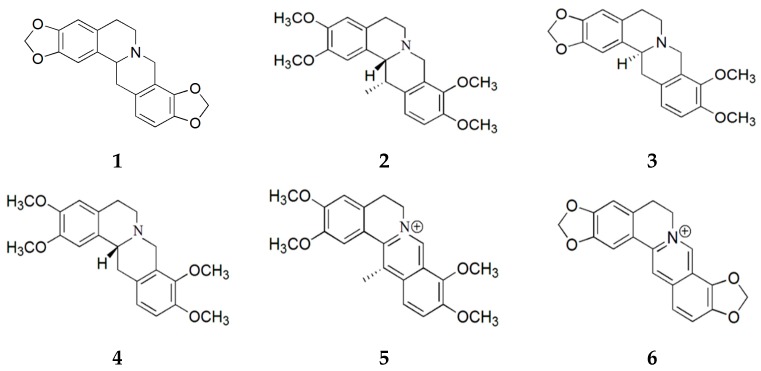
Structures of compounds **1**–**6** isolated from *C. turtschaninovii*.

In this study, we purified six alkaloids from *C. turtschaninovii* (CT) and investigated their myogenic potential in C2C12 myoblasts. Air-dried CT tubers were extracted with aqueous methanol and fractionated to obtain alkaloidal fractions using a conventional method. The alkaloidal fractions were subjected to SiO_2_ column chromatography and six known alkaloids were isolated: stylopine (**1**) [16], corydaline (**2**) [17], canadine (**3**) [18], tetrahydropalmatine (**4**) [19], dehydrocorydaline (**5**) [20], and coptisine (**6**) [20]. Their structures were elucidated by a spectroscopic analysis and a comparison with previously reported data (Figure 1).

### 2.2. Evaluation of Myogenic Activity of CT Compounds

We determined the myogenic effect of six compounds using a reporter gene assay. Since MyoD is a key regulator for the initiation of myogenesis, we measured MyoD transcriptional activity in C2C12 myoblasts expressing the MyoD-responsive reporter 4RTK-luc [21]. The MyoD-responsive reporter 4RTK-luciferase construct contains four E-box sites that are consensus sequences to recognize MyoD, fused to a thymidine kinase promoter. Therefore, luciferase activation means increased MyoD-driven gene expression.

**Figure 2:**
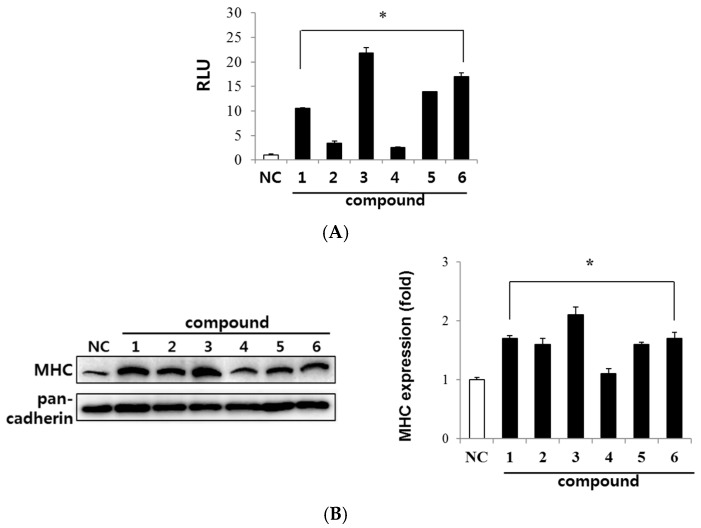
The myogenic effect of CT compounds. (**A**) Effect of the compounds on transactivation of MyoD. C2C12 myoblasts were transiently transfected with MyoD-responsive reporter 4RTK-luciferase (RTK-Luc) vector and pBP-MyoD expression vector. Transfected myoblasts were treated with each compound (1 nM) for 24 h and the cell lysate was subjected to luciferase assay system. Data are presented as relative luciferase activity (RLU) divided by the β-galactosidase activity; (**B**) Effect of compounds on myogenesis. Myoblasts were differentiated in the presence of each compound for 3 days. Protein extracts were analyzed for myosin heavy chain (MHC) expression via Western blotting. Pan-cadherin was used as loading control. Data are expressed as mean ± standard deviation (SD). Images are the representative of three independent experiments that show similar results. NC, negative control; **1**–**6**, compounds **1**–**6**. * *p* < 0.001 vs. NC.

As shown in Figure 2A, all compounds (1 nM) significantly increased the MyoD transcriptional activity as compared with control (compound **1**: 10.5-fold, compound **2**: 3.4-fold, compound **3**: 21.8-fold, compound **4**: 2.5-fold, compound **5**: 13.9-fold, compound **6**: 17.0-fold). Among the tested compounds, canadine (**3**) displayed the highest transcriptional activation of MyoD. Since the expression of myosin heavy chain (MHC) is followed by MyoD activation, MHC is considered as a major marker of myogenesis. We evaluated the effect of the CT compounds on MHC expression by conducting Western blot analysis at 3 days of differentiation. Consistent with MyoD transcriptional activation, all compounds increased MHC expression in differentiated myoblasts (Figure 2B). Canadine (**3**) was the most potent among them.

These results demonstrate that alkaloids from CT have myogenic activity and canadine is the most potent component, showing an increased MHC expression at 1 nM concentration.

### 2.3. Canadine Stimulates Myoblast Differentiation

**Figure 3:**
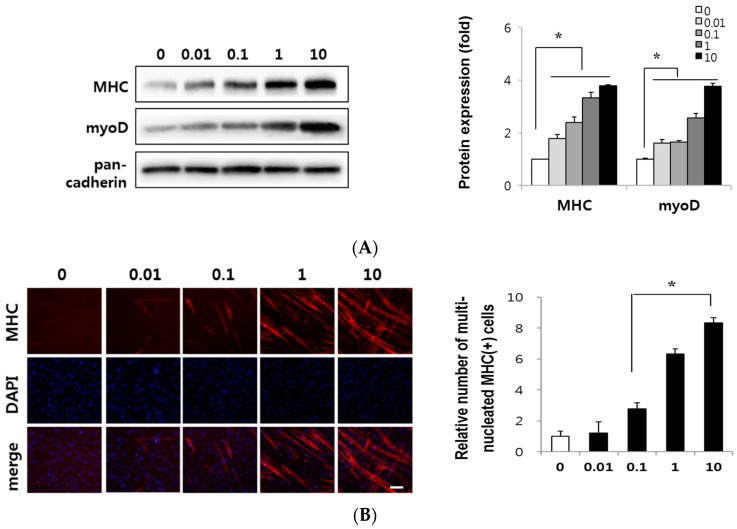
The myogenic activity of canadine. (**A**) Effect of canadine on myogenesis. Myoblasts were differentiated in the presence of canadine (0.01~10 nM) for 3 days. Western blotting was performed to determine the MHC and MyoD expression; (**B**) Effect of canadine on the formation of MHC-expressing multinucleated myotubes. Myoblasts were differentiated as described in Materials and Methods section. Images show the expression of immunofluorescence for MHC (red-colored) and 4′,6-diamidino-2-phenylindole (DAPI) (nuclei, blue-colored) in MHC-expressing multinucleated myotubes. Scale bar = 200 μm. The data present the relative number of MHC-expressing multinucleated cells compared with untreated control group (0 nM). Images are the representative of three independent experiments that show similar results. * *p* < 0.001 vs. 0 nM.

Since canadine showed the most potent MyoD transactivation, we investigated its myogenic effect on C2C12 myoblast differentiation. Myoblasts were differentiated in the presence of canadine (0.01~10 nM) for 3 days and were harvested to conduct Western blot analysis of MHC and MyoD. The treated concentration of canadine showed no change in cell viability or proliferation (data not shown). As shown in Figure 3A, canadine dose-dependently increased the expression of MHC and MyoD up to 3.8- and 3.9-fold at 10 nM, respectively, suggesting its potent myogenic activity.

Confluent C2C12 myoblasts start to differentiate and become more elongated and cylindrical. During myogenesis, these cells fuse together into multinucleated myotubes expressing MHC. Therefore, terminally-differentiated myotubes are multinucleated and express a large amount of MHC.

Differentiated myotubes were immunostained for MHC and counterstained for 4′,6-diamidino-2-phenylindole (DAPI) (Figure 3B). Canadine dose-dependently increased the red-fluorescence intensity indicating the MHC expression in cylinder-shaped myotubes. In addition, MHC-expressing cylinder-shaped myotubes were multinucleated cells when visualized with DAPI staining. The relative number of multinucleated MHC-expressing myotubes increased 8.3 times at 10 nM canadine compared to that of control cells. Based on these results, we could suggest that nanomolar level of canadine is sufficient to induce myoblast differentiation.

### 2.4. Canadine Activates p38 MAPK and Akt Signaling Pathway

Since p38 mitogen-activated protein kinase (p38 MAPK) and Akt serve as key players in the progression of myogenesis [22,23], we evaluated the effect of canadine on the level of phosphorylated p38 MAPK and Akt. p38 MAPK activation is necessary for myogenesis by enhancing MyoD dimerization with E proteins, Mef2 transcription, and chromatin remodeling at muscle-specific genes [24] and blocks premature myogenesis in activated satellite cells [25]. The PI3-kinase/Akt pathway activation is also required for cell survival during myogenesis [26].

**Figure 4:**
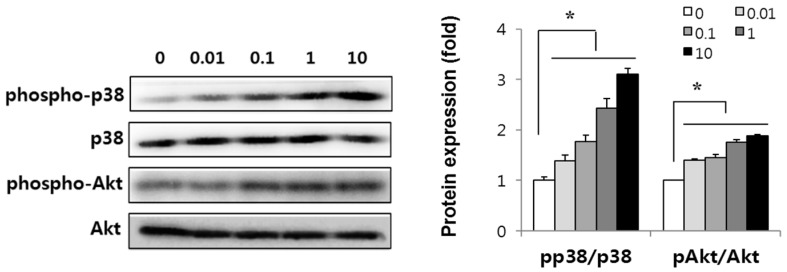
Effect of canadine on p38 MAPK and Akt activation during myogenesis. Myoblasts were treated with canadine (0.01~10 nM) for 3 days and cell lysates were prepared. Protein blots were incubated with an anti-phospho-p38 MAPK or -phospho-Akt antibody. Total p38 MAPK and Akt were used as loading controls. Data represent the mean ± SD of triplicate experiments. * *p* < 0.001 vs. 0 nM.

In this study, we determined whether canadine can activate p38 MAPK and Akt signaling during myoblast differentiation. When we administered 10 nM canadine to C2C12 cells during 3 days’ differentiation, the phosphorylation of p38 MAPK and Akt increased up to 3.2 and 1.9 times, respectively (Figure 4). These results imply that canadine augments myogenesis via p38 MAPK and Akt signaling pathways.

### 2.5. Canadine Prevents Muscle Wasting In Vitro

To evaluate the potential of canadine in overcoming cancer-induced muscle wasting, we treated myotubes with a conditioned medium (CM) of CT26 colon cancer cells. This experimental model was established to mimic cachexia and was widely used to evaluate the protective effect of the chemicals against cancer-induced muscle wasting [6]. Muscle atrophy was observed in CM-treated myotubes due to the increase in inflammatory cytokines including TNF-α, interleukin-6, interleukin-1β and interferon-γ [27].

Considering muscle wasting in the cachexic condition, fully-differentiated myotubes were pre-treated with canadine, and cytokine damage was sequentially induced by CT26 CM. The protection against muscle wasting by canadine was evaluated by measuring the myogenic factor expression and the MHC immunostaining.

**Figure 5:**
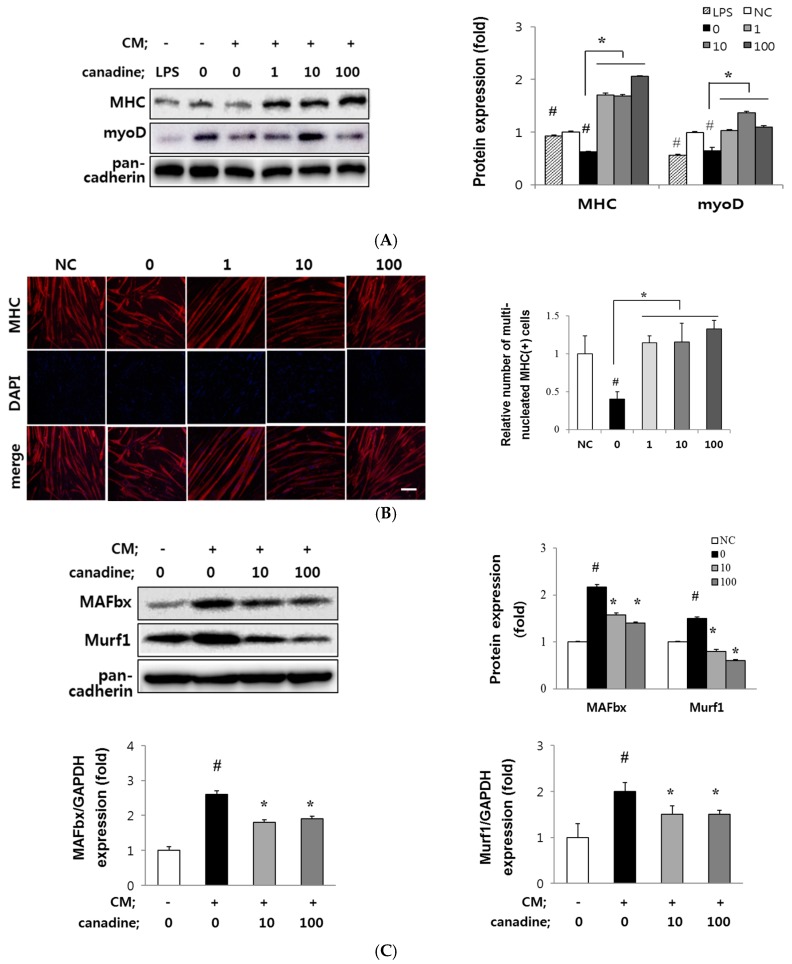
Canadine alleviated CT26 conditioned medium (CM)-induced muscle atrophy in vitro (**A**) Effect of canadine on the expression of MHC and MyoD in CT26 CM-induced muscle wasting. Differentiated myotubes were pre-treated with canadine (1~100 nM) for 3 h, followed by CT26 CM treatment for 24 h. The expression of myogenic markers was determined by Western blot analysis; (**B**) Immunofluorescence for MHC (red) and DAPI (blue) in MHC-expressing multinucleated myotube. Scale bar = 200 μm. Data expressed the relative number of MHC-expressing multinucleated cells compared with the control group (NC). # *p* < 0.001 vs. NC; * *p* < 0.001 vs. CM alone (0 nM); (**C**) Effect of canadine on CM-induced E3 ligase expression. Western blotting was performed to determine the MAFbx and Murf1 expression. Gene expressions of MAFbx and Murf1 were determined using RT-qPCR as described in Materials and Methods. Data are means ± SD of triplicate experiments. # *p* < 0.01 vs. NC; * *p* < 0.01 vs. CM alone (0 nM).

As shown in Figure 5A, CM treatment remarkably decreased MHC and MyoD expression of the myotubes (up to 0.6-fold and 0.6-fold, respectively), indicating CM-induced muscle wasting. Lipopolysaccharide (LPS) was reported as a potent inflammatory agent to mimic cancer-induced muscle wasting [28]. We used LPS as a control to observe the reduced MHC and MyoD protein levels. Pre-treatment with canadine prevented an impairment of MHC and MyoD expression of myotubes in a dose dependent manner (1~100 nM). Immunostaining for MHC also showed protective effect of canadine against muscle wasting. As shown in Figure 5B, myotubes became shorter, and the number of multinucleated MHC-expressing myotubes was reduced under the CT26 CM-damaged condition. Canadine maintained the number of multinucleated myotubes at 100 nM, showing its potential to protect CM-induced muscle wasting.

As further evidence of muscle wasting, we observed the increased expression of muscle specific E3 ligases, such as muscle atrophy F-box (MAFbx/atrogin-1) and Muscle RING finger 1 (Murf1) in CM-treated myotubes. Regarding the preventive effect of canadine on damaged myotubes, pre-treatment with canadine decreased the protein and mRNA levels of MAFbx and Murf1 compared to those in CM-only treated myotubes (Figure 5C).

Based on the above results, canadine protects muscle from atrophy by suppressing E3 ligase expression in CM-induced muscle impairment. As such, canadine can be a preventive agent against muscle atrophy.

## 3. Discussion

The C2C12 cell is an immortal line of mouse skeletal myoblasts derived from mouse satellite cell, which is known as adult muscle stem cell. Myoblasts become myocytes via myogenesis to form muscle fibers in skeletal muscle. After injury, various growth factors, such as hepatocyte growth factor, fibroblast growth factor, and platelet-derived growth factor-BB promote muscle regeneration by inducing the transition of quiescent satellite cells to activated cells, and they start to proliferate and differentiate into myocytes [29]. Myocytes then fuse together or with peripheral myofibers to form multinucleated and cylindrical myotubes during normal muscle regeneration. A series of these processes is referred to as myogenesis.

Well-performing myogenesis is important for effective muscle regeneration to overcome skeletal muscle wasting that might be induced by: (1) diseases including cancer, diabetes, immune deficiency syndrome (AIDS), end stage of renal failure, congestive heart failure, and chronic obstructive pulmonary disease; (2) aging; and (3) atrophy of disuse [5]. More than 30% of patients with cancer die from cachexia (muscle loss generated by diseases), not from the cancer itself. Weight loss in cancer patients contributes to a reduced response to chemotherapy and increased therapy-related side effects, and these lead to a low quality of life in for patients and their families. In an aged population, age-related skeletal muscle loss, known as sarcopenia, is associated with fragility fractures that impede physical activity and independent living for older individuals [30]. Cruz-Jentoft et al. reported that sarcopenia may affect 30% of individuals over 65 years of age, and 50% of those over 80 years of age [31]. With the increase in number of people in the older population with sarcopenia and in the related health care costs, we need an effective treatment for sarcopenia.

The search for therapeutic agents to treat skeletal muscle atrophy has been undertaken for more than two decades. Although several drugs, including eicosapentanoic aicd, β-hydroxy-β-methylbutyrate, ghrelin, and resveratrol have been proposed, the toxicity and side effects resulted in their withdrawal [5]. Therefore, it is urgent to find new agents with less side effects and more efficacious potential to treat muscle atrophy.

As a part of our efforts to discover agents to treat muscle atrophy, we evaluated the myogenic effect of compounds isolated from a medicinal plant, *Corydalis turtschaninovii* (CT) by measuring the level of MHC protein expression and MyoD transactivation during C2C12 cell differentiation. We previously reported on the myogenic potential of isoquinoline alkaloids, tetrahydropalmatine and dehydrocorydaline [12,14]. We further identified myogenic alkaloids including stylopine, corydaline, canadine and coptisine, and canadine showed the strongest myogenic activity among the tested compounds (Figure 2). Canadine has been found in herbal plants [32,33] showing relaxant [34], anti-oxidative [35], anti-platelet aggregation [36], anti-Alzheimer’s disease [37], anti-fibrotic and wound-healing activities [38]. In this study, we first observed the myogenesis-stimulating activity of canadine. As shown in Figure 3, canadine dose-dependently increased MyoD and MHC expression, which was accompanied by cylindrical, multinucleated myotube formation.

The activated p38 MAPK was known to phosphorylate E proteins, Mef2 or SWI/SNF subunit, BAF60 to form a heterodimer of MyoD to induce the expression of essential myogenic factors [25,39]. Moreover, the cross-activation between p38 MAPK and Akt is essential for myogenesis. A complex of p38 MAPK or Akt with scaffold proteins, such as JLP and Bnip-2 for p38 MAPK and APPL-1 for Akt, orchestrates myogenic differentiation. p38 MAPK can also enhance myogenic differentiation via direct activation of Akt [40]. To find whether the p38 MAPK and Akt signaling pathways were involved in canadine-induced myogenesis, we measured the level of phosphorylated p38 MAPK and Akt. Canadine dose-dependently increased p38 MAPK and Akt phosphorylation, indicating that p38 MAPK and Akt activation are at least partly responsible for the myogenesis stimulation by canadine in C2C12 cells. Since the differentiation of myoblasts is essential to form new muscle fibers during muscle regeneration [6], we suggest canadine as a promising agent for treatment of muscle atrophy. We need potential drugs that could improve muscle regeneration by inhibiting the catabolic pathway or by activating the anabolic pathway of muscle proteins to prevent muscle wasting. In muscle wasting conditions, various mediators including myostatin and inflammatory cytokines activate nuclear factor-κB (NF-κB), followed by myofibrillar protein degradation. Ubiquitin-proteasomal degradation of myo-proteins is part of the catabolic machinery in muscle, and it requires the expression of muscle-specific E3 ligase. These include muscle RING finger 1 (MuRF1) [41], muscle atrophy F-box (MAFbx)/atrogin-1 [42], and ubiquitin protein ligase E3 component N-recognin 2 (UBR2) [43]. Therefore, suppressing myo-protein degradation by inhibiting E3 ligase expression may be an effective strategy to prevent or alleviate skeletal muscle wasting.

Conditioned media from CT26 colon carcinoma (CT26 CM) can induce muscle wasting in myotubes, and this in vitro model is well-established to find protective agents against muscle wasting [7,8]. Increased secretion of inflammatory cytokines and E3 ligase expression together with the breakdown of MHC can be markers of muscle wasting in C2C12 myotubes.

In this study, we confirmed that CT26 CM decreased the expression of MHC and MyoD, decreased the number of multinucleated MHC-expressing myotubes, and increased muscle specific E3 ligase expression. The treatment of canadine prior to CT26 CM protected the MHC and MyoD degradation. These data suggested that canadine might have a protective effect against cancer-induced muscle wasting via inhibition of E3 ligase expression (Figure 5). As noted, several proteolytic systems, including the ubiquitin-proteasome system, are responsible for the loss of myo-proteins in muscle. Furthermore, recent studies identified mediators or other pathways contributing to induce skeletal muscle atrophy [44]. Although we found that canadine suppresses the ubiquitin-proteasome system by inhibiting E3 ligase expression, further studies are required to investigate the effect of canadine on other proteolytic system. To investigate a therapeutic effect of canadine, we used fully-differentiated myotubes. Muscle atrophy was induced by treatment of CT26 CM, and the damaged myotubes were incubated with canadine (0–100 nM). Although CT26 CM developed muscle atrophy showing the decreased MHC, canadine could not block the MHC breakdown in myotubes (data not shown).

In recent studies, *Scutellaria baicalensis* and Qing-Shu-Yi-Qi-Tang, a multi-component herbal extract, significantly decreased MuRF-1 expression via NF-κB inhibition and alleviated cachexic symptoms. The extracts increase the therapeutic efficacy and improves the side effects of chemotheraphy in mice challenged with Lewis lung cancer cells [45]. A Kampo formula, Hochuekkito (TJ-41) [46], the natural herb *Coptidis rhizoma* [47], and *Citrus unshiu* peel extract [7] were suggested as promising anti-cachexic agents by reducing the level of inflammatory cytokines in CT26 adenocarcinoma bearing mice. The purified phytochemicals berberine [47], resveratrol [48,49] and curcumin [49] were also reported to exhibit a preventative activity against muscle atrophy through diverse mechanisms.

Taken together, we demonstrate that canadine isolated from *Corydalis turtschaninovii* stimulates myoblast differentiation by promoting the myogenic factor expression via p38 MAPK and Akt activation. Canadine prevents the CM-induced myo-protein degradation by suppressing the muscle-specific E3 ligase expression. Both the stimulation of myogenesis and the inhibition of the myo-proteins degradation under a muscle wasting condition may additively contribute to the preventing effect of canadine on muscle atrophy.

## 4. Materials and Methods

### 4.1. Isolation of Canadine from the Tuber of Corydalis turtschaninovii

*C. turtschaninovii* (CT) tubers were collected at the Kyungdong Herbal Medicine Market (Seoul, Korea) in 2010 and were identified by Professor Dae-Keun Kim (Woosuk University, Jeonju, Korea). A voucher specimen (KHU100012) was reserved at the Laboratory of Natural Products Chemistry (Kyung Hee University, Suwon, Korea). The dried powdered plant materials (1 kg) were extracted in 80% aqueous MeOH (1.3 L × 2) at room temperature overnight, filtered through filter paper, and evaporated in vacuo giving a dark brownish residue. The extract was poured into acidic water (pH 2.5, 500 mL) adjusted using 30% HCl, and washed with EtOAc (500 mL × 2). The aqueous layer was alkalized to pH 11.5 using a 20% NaOH solution and extracted with EtOAc (500 mL × 2). The concentrated organic layer (CTE, 3.6 g) was subjected to column chromatography (4 cm × 6 cm) over SiO_2_ (70–230 mesh, 90 g) using CHCl_3_-MeOH (15:1→13:1→10:1→7:1→5:1→3:1, each 500 mL) and MeOH (1 L) as eluting solvents to give 10 fractions (CTE1~CTE10). The sub-fraction CTE-2 (445 mg, Ve/Vt 0.54–0.68 in CHCl_3_-MeOH = 13:1) was applied to SiO_2_ column chromatography (40 g, 3 cm × 6 cm) using *n*-hexane-EtOAc (1:3, 2 L) as the eluent to yield seven fractions (CTE2-1–CTE2-7) along with canadine [CTE2-5, 88 mg, Ve/Vt 0.72–0.88, TLC (Kiesel gel 60 F_254_) R_f_ 0.37, *n*-hexane-EtOAc = 3:1).

### 4.2. Cell Culture, Myoblast Differentiation and Preparation of Conditioned Medium of Cancer Cells

Mouse C12C12 myoblasts (American Type Culture Collection, Manasas, VA, USA) were cultured and differentiated according to previously-described methods [50]. C2C12 myoblasts were maintained in Dulbecco’s modified Eagle’s medium (DMEM, WelGENE, Daegu, Korea) containing 15% fetal bovine serum (FBS). For differentiation into myotubes, cells reaching a confluence of 90% were cultured in differentiation medium (DM, 2% horse serum-containing DMEM) until myotube formation was observed (normally at 3 days of differentiation).

For the in vitro muscle wasting experiments, differentiated myotubes were pre-treated with canadine for 3 h and were supplemented with CT26-conditioned media (CT26 CM) diluted in DM (30%). After three days, the cells were harvested to conduct a Western blotting analysis and quantitative real-time reverse transcription polymerase chain reaction (RT-qPCR).

To prepare the cancer cell-conditioned medium, CT26 murine colon carcinoma cells (kindly provided by Prof. M.K. Sung, Sookmyung Women’s University, Seoul, Korea) were maintained in growth media (10% FBS-containing DMEM). For CM collection, CT26 cells were plated in 100-mm culture dishes at a density of 1.5 × 10^6^ and incubated in growth media for 24 h. After washing with phosphate-buffered saline (PBS), the cells were replaced with serum free DMEM and incubated for 24 h to exclude the serum inflammatory factors. The resulting CM was centrifuged and filtered using a 0.22 μm syringe filter, followed by dilution with myoblast DM.

### 4.3. MyoD-Reporter Gene Assay

C2C12 cells were transiently transfected with plasmids, MyoD-responsive reporter 4RTK-luciferase (RTK-Luc) and pBP-MyoD using Lipofectamine^®^ 2000 Reagent (Invitrogen, Carlsbad, CA, USA). The RTK-Luc construct, which contains four E-box sites fused to a thymidine kinase promoter was used to analyze the MyoD transcriptional activity [51]. After 24 h of reporter gene transfection, the transfected cells were treated with test compound for 24 h. The cell lysate was subjected to luciferase assay system (Promega, Madison, WI, USA). Data are reported as relative luciferase activity (RLU) divided by β-galactosidase activity.

### 4.4. Immunostaining for MHC

Differentiated myotubes were fixed with 4% paraformaldehyde for 20 min and permeabilized with 0.1% Triton X-100 (Sigma-Aldrich, St. Louis, MO, USA) for 30 min. After washing with PBS, the cells were incubated overnight at 4 °C with a primary antibody specific to MHC (MAB4470, R&D Systems, Minneapolis, MN, USA). Then, the cells were labelled with a goat anti-mouse antibody conjugated with Alexa Fluor 568 (LifeTechnologies, Carlsbad, CA, USA). In addition, the cells were counterstained with DAPI (4′,6-diamidino-2-phenylindole, Sigma-Aldrich), and the MHC immunofluorescence was detected using a fluorescence microscope (Olympus, Tokyo, Japan). A red fluorescence indicates MHC expression, and the multinucleated myotubes are observed with DAPI (blue-colored) counterstaining. The degree of MHC-expressing multinucleated myotubes was presented as a relative change to that of control group.

### 4.5. Western Blot Analysis

To investigate the effect of canadine on myogenesis and muscle wasting, the protein levels of the myogenic markers, phospho-p38 MAPK and -Akt were analyzed using Western blotting analysis. The total protein was electrophoresed in SDS-polyacrylamide gels and transferred to polyvinylfluoride membranes. The membrane was probed with primary antibodies against MHC (sc-376157, Santa Cruz, Dallas, TX, USA, 1:1000), MyoD (sc-32758, 1:1000), myogenin (sc-12732, 1:1000), phospho-p38 MAPK (9211, Cell Signaling Technology, Danvers, MA, USA, 1:1000), p38 MAPK (9212, 1:1000), phospho-Akt (9271, 1:1000), or AKT (9272, 1:1000). Pan-cadherin (C3678, Sigma, St. Louis, MO, USA, 1:2000) was used as a loading control for myogenic markers. Total p38 MAPK and Akt served as control for phospho-p38 MAPK and -Akt expression. The protein levels were quantified using the Fusion Solo system (Vilber Lourmat, Collegien, France). Secondary goat anti-mouse IgG-HRP (sc-2005, 1:5000) and goat anti-rabbit IgG-HRP (sc-2004, 1:5000) were purchased from Santa Cruz.

### 4.6. RNA Extraction and RT-qPCR

To estimate the effect of canadine on the gene expression of E3 ligases, the myotubes were pre-incubated with canadine prior to CT26 CM treatment, as described above. RNA purification from the cells and first-strand cDNA synthesis were performed according to the manufacturer’s instructions (Labopass™ cDNA synthesis kit, Cosmogenetech, Seoul, Korea). An RT-qPCR reaction was performed with the SYBR^®^ Green PCR Master Mix and conducted using the Applied Biosystems 7500 Fast Real-Time PCR System (Foster City, CA, USA). All mRNA levels were normalized using glyceraldehyde 3-phosphate dehydrogenase mRNA as an internal control. The primers used for the amplifications are shown in Table 1.

### 4.7. Statistical Analysis

Data are expressed as mean ± standard deviation and differences were assessed using Student’s *t*-test or one-way ANOVA followed by Dunnett’s test. All experiments were conducted at least three times. A *p*-value < 0.05 was considered significant.

## Figures and Tables

**Table 1:** Oligonucleotide primer sequences used for the qRT-PCR analysis.

Gene Name	Forward Primer	Reverse Primer	Accession Number
*MAFbx*	CGACCTGCCTGTGTGCTTAC	CTTGCGAATCTGCCTCTCTG	BC027211
*Murf1*	GGTGCCTACTTGCTCCTTGT	CTGGTGGCTATTCTCCTTGG	NC_000070
*GAPDH*	TGCACCACCAACTGCTTAG	GGCATGGACTGTGGTCATGAG	BC096042

MAFbx, muscle atrophy F-box (MAFbx/atrogin-1); Murf1, muscle RING finger 1; GAPDH, glyceraldehyde 3-phosphate dehydrogenase.
